# Medication-related knowledge and behavior profiles are generally favorable, but counseling needs remain among pregnant and postpartum women in Taiyuan, China: a cross-sectional study

**DOI:** 10.3389/fpubh.2026.1969907

**Published:** 2026-10-16

**Authors:** Chaolong Zhang, Yingju He, Yuanyuan Yang, Li Zhang, Wenfei Zhang, Liuye Yang, Mengyao Ma

**Affiliations:** 1 Department of Pharmacy, Taiyuan Taihang Hospital, Taiyuan, China; 2 Department of Obstetrics, Taiyuan Taihang Hospital, Taiyuan, China; 3 Department of Operation Management, Taiyuan Taihang Hospital, Taiyuan, China; 4 Department of Pharmacy, Shanxi Provincial People’s Hospital, Taiyuan, China

**Keywords:** knowledge-attitude-practice, medication safety, medication-use risk, pharmaceutical counseling, postpartum period, pregnancy

## Abstract

**Objective:**

To characterize medication-related Knowledge-Attitude-Practice (KAP) risk profiles, pregnancy- and lactation-specific medication familiarity, and demographic correlates among pregnant and postpartum women in Taiyuan, China.

**Methods:**

We conducted a hospital-based cross-sectional survey at Taiyuan Taihang Hospital from July to October 2024 using convenience sampling and an adapted medication-related KAP questionnaire. Higher knowledge-, attitude-, and behavior-risk scores indicated less favorable profiles, whereas higher familiarity scores indicated greater self-rated familiarity. Internal consistency was assessed using Cronbach’s α. Associations were examined using Pearson correlations, Welch’s analysis of variance with Games-Howell *post hoc* tests, and multivariable ordinary least-squares regression with HC3 robust standard errors.

**Results:**

Of 500 returned questionnaires, 497 were analyzed. Cronbach’s α ranged from 0.808 to 0.957. Mean knowledge-risk and behavior-risk scores were 59.78 ± 18.99 and 57.22 ± 11.29, respectively, both within the prespecified good category; the mean attitude-risk score was 37.34 ± 7.94, within the pass category. For attitude risk, 59.0% were classified as pass, 16.1% as fail, and 24.9% as good or excellent. Knowledge risk was inversely correlated with attitude risk (*r* = −0.258) and positively correlated with behavior risk (*r* = 0.275), while attitude risk was weakly positively correlated with behavior risk (*r* = 0.118; all *p* ≤ 0.009). In the adjusted behavior-risk model, higher scores were observed among participants aged 35–49 years and those with other insurance or self-payment. The overall adjusted association between monthly income and behavior risk was not statistically significant (Wald *p* = 0.098); an isolated contrast between the 4,000–6,000-yuan and below-2,000-yuan groups reached nominal statistical significance and was treated as exploratory. Familiarity scores across five pregnancy- and lactation-specific topics ranged from 2.46 to 2.60 on a five-point scale.

**Conclusion:**

Favorable knowledge- and behavior-risk profiles coexisted with less favorable attitude-risk classifications and limited topic-specific familiarity. Broad medication-safety counseling should be integrated into antenatal and postpartum care, with proactive medication review considered for women aged 35–49 years, those with other insurance or self-payment, and those with complex medication needs. The income finding should be regarded as exploratory. Multicenter prospective studies are needed to assess generalizability and evaluate this service approach.

## 1 Introduction

Self-medication refers to the selection and use of medicines for illnesses or symptoms identified by the individual. It also includes continued or intermittent use of a previously prescribed medicine for a chronic or recurrent condition (1). For pregnant and postpartum women, however, the relevant safety questions are not limited to whether a medicine was prescribed. Treatment may need to be started, continued, adjusted, or discontinued. At the same time, women may use over-the-counter products, traditional medicines, or supplements for pregnancy-related symptoms, acute illness, or chronic disease. Pharmacokinetic changes during pregnancy can alter drug exposure and response (2, 3), affecting maternal efficacy and toxicity as well as fetal or breastfeeding exposure (4). Avoiding medication altogether can also cause harm if necessary treatment is delayed or inadequate. Safe use therefore requires a decision based on the woman’s clinical circumstances and the expected benefits and risks of the available options.

The questions that women face also change over time. Pregnancy, delivery, discharge, breastfeeding, and postpartum recovery may each bring a different treatment decision. Antenatal clinics, maternity services, community health services, and pharmacies consequently encounter women at several points when medicines can be reviewed and safety concerns discussed. General education delivered once may not address later questions about gestational hypertension or hyperglycemia, postpartum analgesia, lactation suppression, or treatment while breastfeeding. The “Healthy China 2030” strategy places maternal care within a life-course approach to health services (5), while national policy calls for integrated care during pregnancy and the postpartum period (6). Evidence from local patients is needed to determine what medication support would be useful within these services.

Results from other settings show that medication knowledge, attitudes, and practice do not always agree. In western Uganda, 42.6% of pregnant women had adequate knowledge and 34.5% demonstrated rational medication practice despite generally positive attitudes (7). Information, acceptance of safety education, and application of advice are related, but they describe different aspects of medication use. A national health-insurance database has documented patterns of medication use during pregnancy in mainland China, including potentially higher-risk exposure (8). Those data describe which medicines were used. They do not show how women understood the risks or reached treatment decisions. Studies of knowledge, attitudes, and practice can address this different question.

The Knowledge-Attitude-Practice (KAP) framework is used in public health to examine what people know, believe, and do about a health issue and to guide the planning and evaluation of interventions (9, 10). For medication safety, a favorable total score can conceal uncertainty about a particular decision or disagreement among domains. Risk-oriented scoring places unfavorable responses in the same numerical direction while retaining separate knowledge, attitude, and behavior scores. Their correlations show whether the domains vary together in the sample, although a cross-sectional survey cannot establish the order in which any changes occurred. Questions about familiarity with specific medication topics may reveal counseling needs that broad domain totals miss.

Little is known about medication-related KAP among pregnant and postpartum women receiving care in Taiyuan. Access to pharmaceutical services, health literacy, insurance, employment, and place of residence could contribute to differences within this population. A sample from one hospital cannot represent all women in Taiyuan or elsewhere in China. It can nevertheless describe the patients surveyed, reveal questions that require further study, and help shape a multicenter investigation. The results can also indicate whether counseling needs appear widespread or concentrated around particular topics and participant characteristics.

This study used a risk-oriented KAP questionnaire among pregnant and postpartum women attending a hospital in Taiyuan. We characterized knowledge-, attitude-, and behavior-risk profiles and examined the internal consistency and correlations of the three domains. We also measured self-rated familiarity with five medication topics specific to pregnancy and lactation and explored demographic factors associated with knowledge- and behavior-risk scores. The purpose was to identify counseling needs in this setting and develop questions for future service research. The design was not intended to estimate population prevalence, determine causation, or evaluate an intervention.

## 2 Materials and methods

### 2.1 Study design and participants

This hospital-based cross-sectional questionnaire survey was carried out at Taiyuan Taihang Hospital between July and October 2024. We used convenience sampling to recruit pregnant and postpartum women aged 19 years or older from the hospital’s outpatient and inpatient departments. After agreeing to participate, each eligible woman completed an anonymous questionnaire on site and returned it directly to the study team. Investigators assisted only those participants who could not complete the questionnaire without help.

The Medical Ethics Committee of Taiyuan Taihang Hospital approved the study (approval No. YXLL-2024-001). All participants gave informed consent before taking part in any study procedure. The study followed the Declaration of Helsinki.

### 2.2 Questionnaire and data collection

#### 2.2.1 Questionnaire development and administration

We adapted a questionnaire developed by the Science and Technology Development Center of the Chinese Pharmaceutical Association. The instrument was revised to cover medication use during pregnancy and the postpartum period (Supplementary material). Before data collection, investigators completed standardized training and passed an assessment. They distributed and collected the questionnaires on site after obtaining informed consent.

Each returned questionnaire was checked for completeness and logical consistency. The project lead reviewed forms with substantial missing data, implausible response patterns, or the same response across items before excluding them.

#### 2.2.2 Questionnaire content

The questionnaire contained 76 items: 8 demographic items, 28 medication-knowledge risk items (Q1-Q28), 24 medication-behavior risk items (Q29-Q52), 11 attitude-related items (Q53-Q63), and 5 self-rated familiarity items addressing medication use during pregnancy and lactation (Q64-Q68). The attitude-related items assessed exposure to medication education and support for rational-medication education. Demographic variables included sex, age, monthly income, residential area, medical insurance, education, work status, and occupation. Because all eligible participants were women, sex had no variability and was excluded from descriptive comparisons and regression analyses.

#### 2.2.3 Quality control and psychometric assessment

Data quality was supported by standardized investigator training, on-site completeness checks, double review, and double data entry. Domain-specific valid sample sizes could vary when item responses were missing or excluded during quality control.

Internal consistency was evaluated using Cronbach’s α. Items 32, 33, 35–39, and 45 were reverse-scored before the behavior-domain total was calculated. Cronbach’s α values of 0.70 or higher were considered acceptable. We estimated 95% confidence intervals using 2,000 bootstrap resamples.

We additionally conducted an exploratory assessment of structural validity. The knowledge-risk items (Q1-Q28), behavior-risk items (Q29-Q52), and attitude-related items (Q53-Q63) were analyzed separately. Because responses were ordinal five-category variables, exploratory factor analyses were based on polychoric correlation matrices. Sampling adequacy was assessed using the Kaiser-Meyer-Olkin (KMO) measure and Bartlett’s test of sphericity. The number of factors to retain was determined using Horn’s parallel analysis with 5,000 random replications. Principal-axis factoring with oblique rotation was then used because correlations among latent dimensions were expected. Factor loadings, cross-loadings, communalities, and corrected item-total correlations were examined together with the conceptual content of the items. The exploratory analyses were intended to characterize the empirical structure of the item sets rather than to establish definitive construct validity.

#### 2.2.4 Questionnaire scoring standards

Each KAP item was rated on a five-point Likert scale. Knowledge and behavior items were coded so that higher domain totals indicated greater medication-related risk. To align score direction across domains, the raw positive-attitude total was reverse-transformed as 66 minus the raw total; higher attitude-risk scores therefore indicated a less favorable profile. The five familiarity items were scored from 1 (very unfamiliar) to 5 (extremely familiar), with higher scores indicating greater self-rated familiarity.

The prespecified score categories are listed in Table 1. For knowledge risk, scores of 28–56 denoted excellent, 57–84 good, 85–112 pass, and 113 or higher fail. The corresponding categories for attitude risk were 11–22, 23–33, 34–44, and 45 or higher. Behavior-risk scores were categorized as excellent at 24–48, good at 49–72, pass at 73–96, and fail at 97 or higher. Thus, a lower score in any of the three risk domains represented a more favorable medication-related KAP profile.

**Table 1:** Prespecified classification of medication-related KAP risk scores.

KAP score	Evaluation standard			
	Excellent	Good	Pass	Fail
Total medication knowledge risk score	28–56	57–84	85–112	≥113
Total medication attitude risk score	11–22	23–33	34–44	≥45
Total medication behavior risk score	24–48	49–72	73–96	≥97

### 2.3 Statistical analysis

We described categorical variables using frequencies and percentages and continuous variables using means and standard deviations. Cronbach’s α was calculated for internal consistency; its 95% confidence intervals were based on 2,000 bootstrap resamples. Domain scores were calculated only when all items in the corresponding domain were available. Analyses involving multiple domain scores or covariates were restricted to complete cases.

Exploratory structural analyses were performed separately for the knowledge-risk, behavior-risk, and attitude-related item sets. Polychoric correlation matrices were used because questionnaire responses were ordinal. Factorability was evaluated using the KMO statistic and Bartlett’s test of sphericity. Horn’s parallel analysis based on 5,000 random replications was used to determine the number of factors to retain. Principal-axis factoring with oblique rotation was used to estimate the factor solutions. Residual fit was summarized using the root mean square residual (RMSR). Corrected item-total correlations were also examined.

Pearson correlation coefficients were used to examine associations among knowledge-risk, attitude-risk, and behavior-risk scores. Differences in domain scores across demographic groups were assessed using Welch’s analysis of variance (ANOVA); for variables with two categories, this is equivalent to Welch’s independent-samples t test. Games-Howell *post hoc* comparisons were conducted when an overall test involving three or more groups was statistically significant.

A multivariable ordinary least-squares regression model was fitted for the behavior-risk score. Age, monthly income, residential area, medical-insurance status, education level, work status, and healthcare-related occupation were entered simultaneously. Occupation was analyzed as a nine-category variable in univariable analyses and dichotomized as medical-institution staff versus all other occupations in the multivariable model. HC3 heteroskedasticity-consistent standard errors were used to account for potential heteroskedasticity. We report unstandardized coefficients (B), robust 95% confidence intervals, and two-sided *p* values. An overall Wald test assessed the association between monthly income and behavior-risk score. Statistical significance was defined as *p* < 0.05.

Analyses were performed in Python 3.13.5. Welch’s ANOVA and Games-Howell *post hoc* comparisons were implemented with SciPy 1.17.0, and ordinary least-squares regression with HC3 robust standard errors was performed with Statsmodels 0.14.6.

## 3 Results

### 3.1 Participant characteristics

Of 520 questionnaires distributed, 500 were returned (response rate, 96.2%). Three were excluded during data-quality review, leaving 497 participants for analysis; 99.4% of returned questionnaires were retained. Most participants were aged 19–34 years (68.2%), lived in urban areas (72.4%), had basic social medical insurance (78.9%), had a college/bachelor’s or postgraduate education (83.3%), and were employed (79.5%). The largest monthly-income group was 2,000–4,000 yuan (33.8%). The most common occupational categories were company employees (20.5%), enterprise workers (17.1%), medical-institution staff (12.9%), freelancers (12.5%), and other occupations (19.9%) (Table 2).

**Table 2:** Demographic characteristics of participants.

Variable	Category	*n* (%)
Age	19–34 years	339 (68.2)
	35–49 years	158 (31.8)
Income	Below 2000 yuan	84 (16.9)
	2000–4,000 yuan	168 (33.8)
	4,000–6,000 yuan	144 (29.0)
	Above 6,000 yuan	101 (20.3)
Residential area	Urban Residents	360 (72.4)
	Rural Residents	137 (27.6)
Medical insurance status	Social Basic Medical Insurance	392 (78.9)
	Others	105 (21.1)
Education level	Senior High School and Below	83 (16.7)
	Bachelor’s/College Degree	359 (72.4)
	Postgraduate Degree	54 (10.9)
Work status	Employed	395 (79.5)
	Unemployed/Inactive	102 (20.5)
Occupation	Enterprise Worker	85 (17.1)
	Company Employee	102 (20.5)
	Government Official	21 (4.2)
	Medical Institution Staff	64 (12.9)
	Teacher	38 (7.6)
	Enterprise Management	20 (4.0)
	Freelancer	62 (12.5)
	Student	6 (1.2)
	Others	99 (19.9)

Education level was available for 496 participants because one response was missing.

### 3.2 Internal consistency of the questionnaire

Cronbach’s α ranged from 0.808 for the medication behavior-risk domain to 0.957 for the medication knowledge-risk domain; all domains exceeded the prespecified acceptable threshold. Cronbach’s α was 0.895 for the medication attitude-related domain and 0.924 for self-rated familiarity with medication use during pregnancy and lactation (Table 3).

**Table 3:** Internal consistency reliability of the questionnaire domains.

Domain	Items	*n*	Cronbach’s α	95% bootstrap CI
Medication knowledge risk	Q1–Q28	495	0.957	0.944–0.965
Medication behavior risk	Q29–Q52	496	0.808	0.784–0.829
Medication attitude-related domain	Q53–Q63	497	0.895	0.874–0.911
Self-rated familiarity with medication use during pregnancy and lactation	Q64–Q68	497	0.924	0.905–0.938

### 3.3 Exploratory structural validity

The three KAP-related item sets were analyzed separately using polychoric correlations. Sampling adequacy was good for all three domains (KMO = 0.937 for knowledge risk and 0.868 for both behavior risk and the attitude-related domain), and Bartlett’s tests of sphericity were significant (all *p* < 0.001). Parallel analysis supported three factors for the 28 knowledge-risk items, four factors for the 24 behavior-risk items, and two factors for the 11 attitude-related items. The retained multidimensional solutions substantially reduced the RMSR compared with the corresponding one-factor solutions (knowledge: 0.042 vs. 0.085; behavior: 0.040 vs. 0.182; attitude-related: 0.037 vs. 0.292). The two-factor structure of the attitude-related items was especially interpretable: Q53–Q58 loaded primarily on an education-exposure factor, whereas Q59–Q63 loaded on a perceived-necessity factor. Internal consistency was high for both subdomains (α = 0.925 and 0.948, respectively). Detailed factor loadings are provided in Supplementary Tables 2, 3.

### 3.4 Distribution and correlations of KAP risk scores

#### 3.4.1 Medication knowledge risk

The mean knowledge-risk score was 59.78 ± 18.99 (range, 28–140), within the prespecified good category. Among 495 participants with complete knowledge-domain data, 220 (44.4%) were classified as excellent, 242 (48.9%) as good, 23 (4.6%) as pass, and 10 (2.0%) as fail. Overall, 93.3% were classified as excellent or good (Table 4).

**Table 4:** Distribution of risk-oriented medication-related KAP scores.

Domain	*n*	Mean ± SD	Minimum	Maximum
Medication knowledge risk	495	59.78 ± 18.99	28	140
Medication attitude risk	497	37.34 ± 7.94	11	55
Medication behavior risk	496	57.22 ± 11.29	28	88

#### 3.4.2 Medication attitude risk

The mean reverse-oriented attitude-risk score was 37.34 ± 7.94 (range, 11–55), within the pass category. Of 497 participants, 26 (5.2%) were classified as excellent, 98 (19.7%) as good, 293 (59.0%) as pass, and 80 (16.1%) as fail (Table 4).

#### 3.4.3 Medication behavior risk

The mean behavior-risk score was 57.22 ± 11.29 (range, 28–88), within the good category. Among 496 participants with complete behavior-domain data, 119 (24.0%) were classified as excellent, 342 (69.0%) as good, and 35 (7.1%) as pass; none were classified as fail. Overall, 92.9% were classified as excellent or good (Table 4).

#### 3.4.4 Correlations among KAP domains

Pearson correlations among the three KAP risk scores are shown in Table 5. Knowledge risk was inversely correlated with attitude risk (*r* = −0.258, *p* < 0.001) and positively correlated with behavior risk (*r* = 0.275, *p* < 0.001). Attitude risk was weakly positively correlated with behavior risk (*r* = 0.118, *p* = 0.009).

**Table 5:** Pearson correlations among medication-related KAP domain scores.

Domain	Knowledge risk	Attitude risk	Behavior risk
Knowledge risk	1.000	−0.258**	0.275**
Attitude risk	−0.258**	1.000	0.118**
Behavior risk	0.275**	0.118**	1.000

Complete-case *n* = 494. **p < 0.01.

### 3.5 Self-rated familiarity with medication use during pregnancy and lactation

Across the five self-rated familiarity topics, mean scores ranged from 2.46 to 2.60 (Table 6). Figure 1 shows the response distributions across the five familiarity categories. For each topic, “unfamiliar” and “generally familiar” were the most common responses, whereas “extremely familiar” was the least common.

**Table 6:** Self-rated familiarity with medication use during pregnancy and lactation.

Familiarity topic	Mean ± SD
Do you understand the risk classification of drug use during pregnancy and lactation	2.60 ± 1.04
Do you have knowledge about medications for gestational hypertension and gestational hyperglycemia	2.52 ± 1.03
Do you understand the relevant medication use during the postpartum lactation period	2.54 ± 1.05
What do you know about medications for postpartum lactation suppression	2.46 ± 1.07
Do you understand postpartum analgesic medications	2.48 ± 1.09

**Figure 1:**
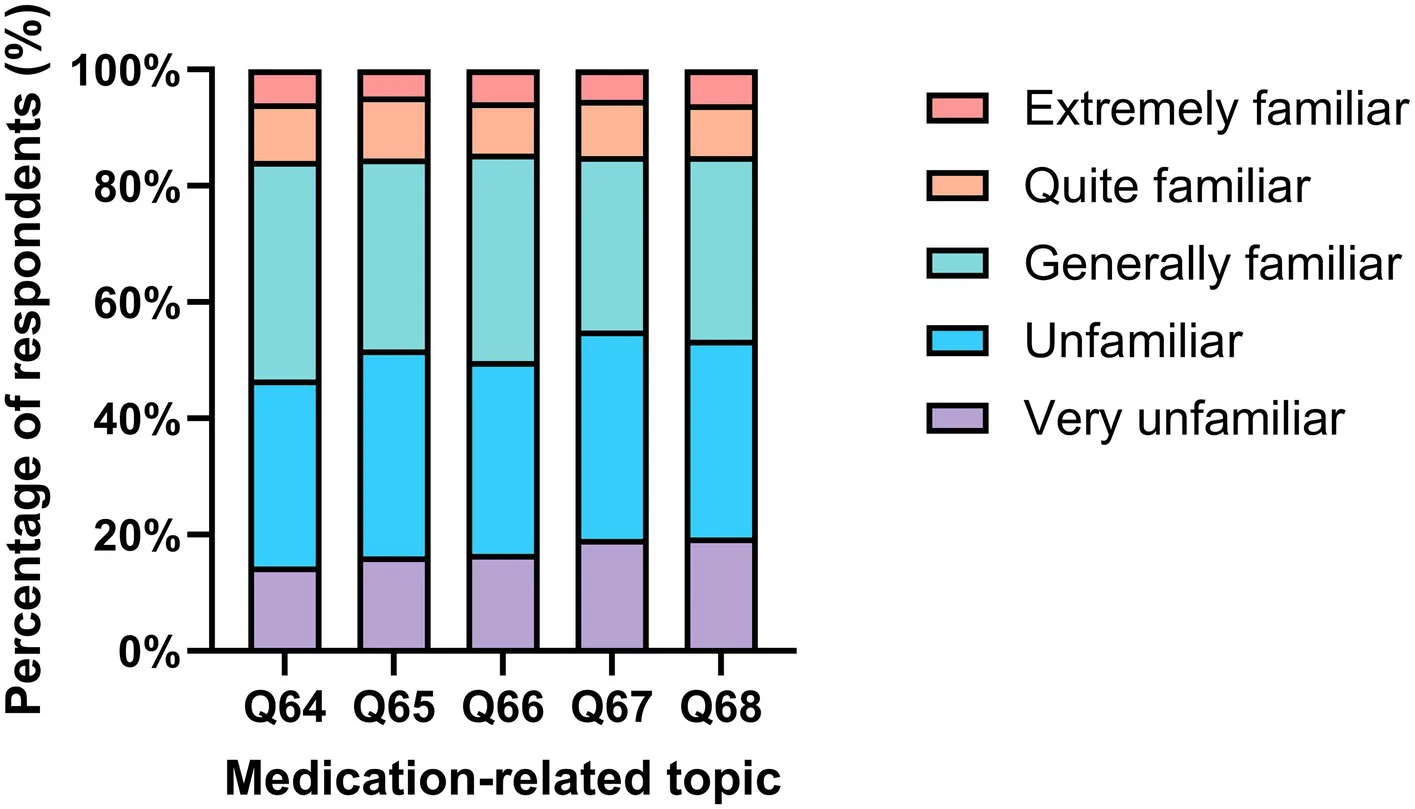
Self-rated familiarity with medication use during pregnancy and lactation among pregnant and postpartum women (100% stacked bar chart, *n* = 497).

### 3.6 Univariable analyses and multivariable regression

In univariable analyses, knowledge-risk scores differed by monthly income (*p* = 0.017), residential area (*p* = 0.021), work status (*p* = 0.018), and occupation (*p* = 0.027). The lowest mean knowledge-risk score was observed among participants earning above 6,000 yuan. Urban residents had lower mean scores than rural residents (58.48 ± 18.11 vs. 63.17 ± 20.81), and employed participants had lower mean scores than unemployed/inactive participants (58.85 ± 19.54 vs. 63.35 ± 16.32). No demographic variable was associated with attitude-risk scores (all *p* > 0.05) (Table 7).

**Table 7:** Univariable analysis of KAP risk scores by participant characteristics.

Influencing factor	Knowledge		Attitude		Behavior	
	Mean ± SD	*p*	Mean ± SD	*p*	Mean ± SD	*p*
Age						
19–34 years	59.94 ± 18.46	0.796	36.96 ± 7.74	0.127	56.13 ± 11.37	0.001
35–49 years	59.44 ± 20.15		38.16 ± 8.33		59.57 ± 10.77	
Income						
Below 2000 yuan	63.20 ± 20.86	0.017	36.86 ± 9.53	0.869	55.66 ± 13.42	0.077
2000–4,000 yuan	59.18 ± 16.54		37.66 ± 6.38		57.17 ± 11.64	
4,000–6,000 yuan	61.45 ± 22.32		37.43 ± 8.49		59.01 ± 10.00	
Above 6,000 yuan	55.56 ± 15.05		37.07 ± 8.12		56.03 ± 10.28	
Residential area						
Urban residents	58.48 ± 18.11	0.021	37.24 ± 7.83	0.660	57.45 ± 10.57	0.503
Rural residents	63.17 ± 20.81		37.60 ± 8.24		56.61 ± 13.02	
Medical insurance status						
Social basic medical insurance	59.21 ± 18.84	0.211	37.35 ± 7.83	0.942	56.42 ± 11.22	0.002
Others	61.89 ± 19.49		37.29 ± 8.38		60.22 ± 11.09	
Education level						
Senior high school and below	62.64 ± 22.54	0.252	37.30 ± 9.02	0.890	56.96 ± 11.78	0.861
Bachelor’s/College degree	59.27 ± 18.12		37.46 ± 7.85		57.12 ± 11.49	
Postgraduate degree	57.26 ± 15.12		37.00 ± 6.29		57.78 ± 8.48	
Work status						
Employed	58.85 ± 19.54	0.018	37.19 ± 8.01	0.411	57.32 ± 11.17	0.688
Unemployed/Inactive	63.35 ± 16.32		37.90 ± 7.68		56.80 ± 11.77	
Occupation						
Enterprise worker	59.04 ± 20.13	0.027	37.84 ± 9.15	0.449	56.99 ± 11.59	0.562
Company employee	60.77 ± 21.21		37.53 ± 8.44		56.96 ± 10.72	
Government official	55.67 ± 11.79		37.86 ± 6.11		58.62 ± 8.04	
Medical institution staff	56.14 ± 17.71		35.31 ± 7.76		57.42 ± 11.76	
Teacher	57.29 ± 19.53		36.84 ± 8.22		59.34 ± 10.20	
Enterprise management	52.58 ± 11.51		39.40 ± 6.50		54.85 ± 9.39	
Freelancer	59.85 ± 17.13		38.40 ± 7.56		58.61 ± 11.87	
Student	60.83 ± 11.77		35.67 ± 10.48		61.67 ± 9.31	
Others	64.86 ± 19.27		37.12 ± 6.93		55.77 ± 12.35	

*p* values were obtained using Welch’s analysis of variance.

Behavior-risk scores differed by age (*p* = 0.001) and medical-insurance status (*p* = 0.002). Participants aged 35–49 years had a higher mean score than those aged 19–34 years (59.57 ± 10.77 vs. 56.13 ± 11.37), and participants with other insurance or self-payment had a higher mean score than those with basic social medical insurance (60.22 ± 11.09 vs. 56.42 ± 11.22) (Table 7).

Games-Howell *post hoc* comparisons are shown in Table 8. Participants earning below 2,000 yuan had higher knowledge-risk scores than those earning above 6,000 yuan (adjusted *p* = 0.029). Participants in the “other occupations” category had higher scores than those in enterprise management (adjusted *p* = 0.015). Only statistically significant pairwise comparisons are shown in Table 8; complete *post hoc* results are provided in Supplementary Table 1.

**Table 8:** Significant Games-Howell *post hoc* comparisons of medication-knowledge risk scores by monthly income and occupation.

Factor	Significant pairwise comparison	Group means (x̄ ± s)	Mean difference (95% CI)	Adjusted *p* value
Monthly income	Below 2,000 yuan (*n* = 84) vs. above 6,000 yuan (*n* = 101)	63.20 ± 20.86 vs. 55.56 ± 15.05	7.64 (0.56 to 14.72)	0.029
Occupation	Other occupations (*n* = 99) vs. enterprise management (*n* = 19)	64.86 ± 19.27 vs. 52.58 ± 11.51	12.28 (1.55 to 23.01)	0.015

Group-specific sample sizes varied because knowledge-risk scores were unavailable for two participants.

In the multivariable behavior-risk model, age 35–49 years was associated with higher behavior-risk scores (*p* = 0.003), and other insurance/self-payment was associated with higher scores than basic social medical insurance (*p* = 0.002). The 4,000-6,000-yuan income category also had a higher adjusted score than the below-2,000-yuan reference category (*p* = 0.047). However, the overall Wald test for monthly income was not statistically significant (*p* = 0.098). Residence, education, work status, and healthcare-related occupation were not independently associated with behavior risk (Table 9).

**Table 9:** Multivariable linear regression for medication-behavior risk score.

Predictor	Reference category	B	*p*	95% CI
Age: 35–49 years	19–34 years	3.202	0.003	1.119 to 5.285
Monthly income: 2,000–4,000 yuan	Below 2,000 yuan	2.211	0.228	−1.388 to 5.809
Monthly income: 4,000–6,000 yuan	Below 2,000 yuan	3.584	0.047	0.040 to 7.129
Monthly income: Above 6,000 yuan	Below 2,000 yuan	0.823	0.672	−2.991 to 4.636
Rural residents	Urban residents	−1.119	0.414	−3.806 to 1.568
Other insurance/self-payment	Basic social medical insurance	4.187	0.002	1.602 to 6.772
College/Bachelor’s degree	Senior high school and below	1.112	0.486	−2.021 to 4.245
Postgraduate degree	Senior high school and below	1.269	0.517	−2.578 to 5.116
Unemployed/inactive/retired	Employed	0.872	0.557	−2.043 to 3.787
Healthcare-related occupation	Non-healthcare-related	0.409	0.807	−2.875 to 3.692

Complete-case *n* = 495. B denotes the unstandardized regression coefficient. Confidence intervals and *p* values were calculated using HC3 heteroskedasticity-robust standard errors.

## 4 Discussion

### 4.1 Principal findings

The results did not show uniformly favorable medication-related preparedness. Knowledge-risk and behavior-risk scores fell within the prespecified good range, and more than 90% of participants were classified as excellent or good in each domain. The attitude results differed. The mean attitude-risk score was in the pass range, with 59.0% classified as pass, 16.1% as fail, and 24.9% as good or excellent. Mean familiarity with each of the five pregnancy- and lactation-specific medication topics was below the midpoint of the five-point scale. Thus, favorable results in the knowledge and behavior domains occurred alongside less favorable attitude classifications and limited familiarity with specific medication decisions.

These findings suggest that broad KAP scores and readiness for a particular clinical decision are not interchangeable. A woman may score well on general medication knowledge and behavior yet still need advice when deciding about treatment during pregnancy or lactation. The attitude distribution and the familiarity results point to counseling needs across the sample, with the content adapted to the medication question and clinical stage.

The domain correlations also warrant separate interpretation rather than a presumed progression from knowledge to attitudes and then behavior. Knowledge risk was positively correlated with behavior risk, while attitude risk showed only a weak positive correlation with behavior risk. Knowledge risk and attitude risk were inversely correlated. In the adjusted behavior-risk model, participants aged 35–49 years and those with other insurance or self-payment had higher scores. These associations may help prompt closer medication review, but they do not define who should or should not receive counseling. The contrast between the 4,000–6,000-yuan and below-2,000-yuan income groups should be regarded strictly as exploratory. The omnibus Wald test for monthly income was not statistically significant (*p* = 0.098), and the category-specific means did not show a monotonic dose–response pattern. Accordingly, this isolated contrast does not provide evidence of an independent overall association between income and medication behavior risk.

### 4.2 Interpretation of the KAP profile

Public education on medication safety and rational medicine use has been conducted in China for many years. In 2004, the State Food and Drug Administration launched a nationwide awareness campaign that disseminated information about safe medication use and encouraged consultation with experts (11). This campaign forms part of the setting in which the generally favorable knowledge-risk scores were observed. The present cross-sectional study cannot determine whether the campaign or later education activities produced those scores.

A favorable domain score did not mean that every medication concept was understood. Some participants regarded antibacterial drugs as anti-inflammatory drugs. This specific misconception calls for a focused response: education should distinguish the two concepts, explain bacterial resistance, and discourage inappropriate antibacterial use during pregnancy and the postpartum period.

The attitude findings were less favorable. The mean attitude-risk score was 37.34 ± 7.94, within the pass category, and only 24.9% of participants were classified as good or excellent. Interpretation of this domain is not straightforward because its items covered exposure to medication education as well as support for such education. This interpretation was supported by the exploratory factor analysis, which separated Q53-Q58, reflecting exposure to medication education, from Q59-Q63, reflecting perceived necessity of such education. Most participants considered community lectures on rational medicine use necessary, which is consistent with the demand for information indicated by the familiarity items.

The results also separate general knowledge from access to usable advice. A woman may have a favorable total knowledge score but remain unsure during a time-sensitive medication decision or be unable to obtain consistent professional guidance. Similar concerns were reported in a study of 562 pregnant women in Riyadh. Despite generally favorable KAP responses, more than 58% considered information from physicians inadequate or inconsistent, and 65.7% reported the same concern about pharmacists (12). In both studies, interpretation of total KAP scores needs to account for access to information and decision support.

Item-level behavior responses add detail that the good overall classification does not provide. Some participants used expired medication. Others changed treatment without consulting a healthcare professional when they thought it was ineffective. These practices have different causes and require different responses. Information about storage and disposal is relevant to expired products, whereas unadvised treatment changes point to a need for timely professional consultation. Local education and counseling should therefore be informed by individual responses as well as domain totals.

### 4.3 Relationships among KAP domains

Knowledge risk was positively correlated with behavior risk (*r* = 0.275), but the correlation between attitude risk and behavior risk was weaker (*r* = 0.118). Knowledge and attitudes therefore explained only part of the observed variation in behavior. Other studies have also found that KAP domains do not necessarily move together. In western Uganda, positive attitudes were more common than adequate knowledge or rational practice (7). An Indian study reported high knowledge and favorable attitudes, while only about one third of participants demonstrated rational practice (13). Medication behavior may also depend on access to care, symptoms, established habits, previous treatment, family advice, and contact with healthcare professionals.

Evidence from Indonesia shows why the content of knowledge matters. Among pregnant women receiving primary care in Malang, greater general knowledge of over-the-counter medication was associated with more self-medication. Correct knowledge of possible fetal risks, however, was associated with substantially less self-medication (14). Knowing how to obtain or take a medicine is different from knowing when professional assessment is needed. Education should address decisions and risks that participants are likely to encounter rather than simply increase the amount of information delivered.

In the present study, less favorable knowledge occurred alongside less favorable behavior, as indicated by the positive correlation between the two risk scores. The correlation was modest and is not suitable for predicting an individual’s behavior. Nor can the cross-sectional design show that a change in knowledge would lead to a change in behavior. Medication exposure, chronic disease, opportunities to use medicines, and contact with healthcare services could have affected both scores. Knowledge is consequently one part of medication-safety support, not a sufficient intervention by itself.

The inverse correlation between knowledge risk and attitude risk (*r* = −0.258) is more difficult to explain. Because higher scores indicated greater risk in both domains, participants with higher knowledge risk tended to have lower attitude risk. This does not mean that poorer knowledge was accompanied by less favorable attitudes. One possibility is that participants who were aware of gaps in their knowledge were more receptive to professional guidance. The composition of the attitude domain may also matter because it included medication beliefs, exposure to education, and support for educational activities. Response tendencies, reverse scoring, or the multidimensional structure of the adapted items offer other explanations. The study did not test any of these possibilities.

Further validation should begin with the distribution and factor structure of the attitude items. Comparison with established measures of medication beliefs would help clarify what the domain captures, and subgroup analyses could determine whether its scoring performs similarly in pregnant and postpartum participants. Longitudinal data would be required to establish whether a change in one KAP domain precedes change in another.

### 4.4 Pregnancy- and lactation-specific medication familiarity

Mean familiarity scores were below 3.0 for all five topics. The lowest scores concerned medication for lactation suppression and postpartum analgesia. These needs were not apparent from the overall knowledge-risk score, which illustrates the additional information obtained from topic-specific questions.

The familiarity items measured participants’ own perceptions, not objective knowledge or clinical competence. Pregnant and postpartum participants were also analyzed together. A low score on a postpartum topic could reflect limited education, but it could also reflect clinical stage or previous experience. For example, a participant who had not yet given birth may not have encountered a decision about postpartum analgesia or lactation suppression. Analyses stratified by pregnancy or postpartum status, trimester, parity, breastfeeding status, and previous medication exposure would help distinguish these explanations.

The five topics arise at different points in maternity care. General medication-risk assessment, including the limits of simple risk categories, could be discussed during an antenatal medication review. Treatment for gestational hypertension or hyperglycemia becomes especially relevant when either condition is diagnosed or therapy changes. Breastfeeding-compatible treatment, postpartum analgesia, and lactation suppression may be introduced before delivery and revisited at discharge or follow-up. This study did not compare counseling schedules. It does, however, show a pattern of familiarity that can inform evaluation of education at several clinical transitions rather than at one encounter only.

Counseling needs to explain how maternal benefit is considered alongside fetal or infant risk instead of relying on memorized labels. A Chinese national insurance-database study documented medication exposure during pregnancy, including exposure to some medicines in FDA categories D or X (8). Decisions for an individual patient require assessment of the indication, the medicine, gestational timing, and expected maternal and fetal benefits and risks (15). Pregnancy-related pharmacokinetic changes, dose requirements, and effective alternatives may also be relevant (15). Pharmacists can help clinicians and patients assess the evidence and interpret risk–benefit information during pregnancy and lactation (16). In a quality-improvement initiative, prenatal education about medication and substance exposure during lactation was associated with improved knowledge (17).

### 4.5 Demographic and healthcare-access correlates

In the univariable analyses, knowledge-risk scores differed by income, residence, work status, and occupation. Games-Howell comparisons found two pairwise differences: participants earning below 2,000 yuan versus those earning above 6,000 yuan, and the heterogeneous “other occupations” category versus enterprise management. These comparisons did not produce a coherent demographic pattern. Several occupation groups were small, the “other occupations” category did not represent a common exposure, and the income means did not follow a monotonic trend. The pairwise results should remain exploratory and should not be used to rank demographic groups by knowledge risk.

After adjustment, participants aged 35–49 years had behavior-risk scores 3.20 points higher than those aged 19–34 years. Previous findings on age have not been consistent. An Indonesian study found more self-medication among women aged 28–45 years than among younger women, whereas an Indian KAP study associated older age with better knowledge and more rational medication practices (13, 14). Age alone is therefore unlikely to explain safer or riskier medication behavior across settings. In this sample, it may reflect differences in parity, chronic disease, previous medication experience, symptom burden, or use of multiple medicines. None of these factors was collected. The association can prompt a closer review of medication needs among women aged 35–49 years, but it should not be interpreted as evidence that age caused the higher risk scores. It also identifies variables that should be measured in future studies.

Participants with other insurance or self-payment had adjusted behavior-risk scores 4.19 points higher than participants with basic social medical insurance. Continuity of care, access to prescribing or dispensing services, affordability, and patterns of healthcare use may differ between these groups, but the study did not measure those factors. The result may indicate a healthcare-access dimension that deserves further assessment. In practice, payment status can be one prompt to ask about medication access, continuity of treatment, and unresolved questions. It is not evidence that a particular insurance arrangement directly changes medication behavior.

The income result was less consistent. Although the 4,000–6,000-yuan category differed from the below-2,000-yuan reference category, the omnibus Wald test for monthly income was not statistically significant (*p* = 0.098). Moreover, behavior-risk scores did not show a monotonic dose–response pattern across increasing income categories. The isolated category-specific coefficient should therefore be regarded strictly as an exploratory signal rather than evidence that monthly income independently predicts medication behavior risk. Residence, education, work status, and healthcare-related occupation were not independently associated with behavior risk. Taken together, the results do not justify limiting counseling to narrowly selected demographic groups. Broad access should remain the basis of support, while the observed age and payment-group associations may prompt additional review when considered with an individual’s medication needs.

### 4.6 Implications for public health and pharmaceutical care

Limited familiarity with pregnancy- and lactation-specific topics was found across the sample, and the demographic results did not identify a consistent high-risk group. Medication-safety support should therefore be available broadly through routine antenatal and postpartum care. The intensity of review can then reflect the medication decision and clinical circumstances rather than demographic eligibility alone.

At an initial contact, a clinician or pharmacist could review prescription medicines, over-the-counter products, traditional medicines, and supplements. The review could identify treatment that should not be stopped without clinical assessment and make clear how the patient can obtain professional advice. Teach-back is one option for checking whether the explanation was understood. These suggestions describe a framework for local evaluation. They are not an intervention shown to be effective by the present survey.

The timing of counseling also matters. A medication question may arise when treatment starts or changes, between trimesters, after a pregnancy complication is diagnosed, during admission or discharge, at the beginning of breastfeeding, during postpartum pain management, or when lactation suppression is considered. A short checklist at these contacts could record current medicines, recent self-medication, treatment changes made without consultation, expired products, and unresolved questions. Complex regimens or uncertain risk–benefit decisions would warrant a more detailed pharmacist or multidisciplinary review. Age and payment status may provide additional prompts, but neither should determine access to counseling.

General education and individual counseling address different needs. Public-facing materials can correct common misconceptions and explain when professional assessment is necessary. A consultation can address the indication, timing, breastfeeding, alternatives, expected benefit, and uncertainty in the patient’s own situation. Written and digital resources should direct users to a qualified professional rather than operate as stand-alone lists of medicines to use or avoid.

Earlier interventions offer possible delivery methods, although they do not establish effectiveness in Taiyuan. Medication counseling in a Nepalese pre-post study was associated with improvements in knowledge, attitudes, and practices among pregnant women (18). In Norway, the SafeStart feasibility study found that early-pregnancy consultations with community pharmacists were feasible and highly acceptable. Participants also valued telephone contact and the option of follow-up (19).

A local pilot might combine a brief face-to-face consultation with a pregnancy and lactation medication checklist, decision aids, and telephone or online follow-up. Evaluation should first determine whom the service reaches, whether participants accept it, and whether decision-relevant knowledge and medication behavior change over time. Medication-reconciliation accuracy, non-prescribed medicine use, adherence to necessary treatment, clinically inappropriate exposure, and healthcare use are possible additional outcomes. These are proposed evaluation measures. The self-reported KAP and familiarity data collected in this study cannot show that a counseling program would improve them.

### 4.7 Strengths and limitations

Strengths of this study include a sample of nearly 500 participants, a high questionnaire response rate, and assessment of broad medication-related KAP domains alongside pregnancy- and lactation-specific topics. Internal consistency exceeded the prespecified acceptable threshold across domains, with bootstrap confidence intervals. The analyses used Welch tests and Games-Howell comparisons rather than assuming equal variances, and the multivariable model used HC3 robust standard errors. Consistent risk-oriented scoring across the three KAP domains simplified interpretation of score direction. Reporting attitude classifications and topic-specific familiarity also showed medication-safety needs that were not apparent from the knowledge- and behavior-risk classifications alone.

This study used convenience sampling at a single hospital. Its findings may therefore not extend to women receiving care in other settings or to populations with different demographic characteristics. The questionnaire data were self-reported. Although the adapted instrument showed good internal consistency, its measurement properties still need to be evaluated in other populations. We also analyzed pregnant and postpartum women as one group and did not collect several relevant clinical and medication characteristics. These omissions limited subgroup interpretation. Finally, the cross-sectional data show associations at one point in time and cannot establish their temporal order or causality.

Further work should recruit women from several hospitals and community settings, with separate analyses for pregnant and postpartum participants. Collecting more detailed clinical and medication information would allow the observed associations to be examined with less residual uncertainty. The questionnaire also requires further validation, including assessment of its performance in different subgroups. Survey scores could be compared with prescription, dispensing, or medication-reconciliation records to determine how closely self-reported risk corresponds to observed medication use. A prospective design would then be needed to assess whether staged counseling changes decision-relevant knowledge or medication behavior. Such studies could also test whether the service approach proposed here is transferable beyond the present setting.

## 5 Conclusion

Among women surveyed at this hospital, medication knowledge- and behavior-risk profiles were generally favorable, but attitude-risk classifications and limited familiarity with pregnancy- and lactation-specific topics revealed continuing counseling needs. Overall KAP classifications did not necessarily indicate that participants were prepared for individual medication decisions. The results support offering medication-safety counseling during routine antenatal and postpartum care, with advice adapted as medication needs change. More proactive review may be appropriate for complex regimens. Participants aged 35–49 years and those with other insurance or self-payment had higher adjusted behavior-risk scores, although these associations should be treated as signals for further assessment rather than fixed targeting criteria. The isolated income-category contrast remains exploratory because the overall association of monthly income with behavior risk was not statistically significant and no monotonic dose–response pattern was observed across income categories. Multicenter prospective studies are needed to determine whether the findings are generalizable and whether staged counseling improves decision-relevant knowledge and medication behavior.

## Data Availability

The raw data supporting the conclusions of this article will be made available by the authors, without undue reservation.
