# Supplementary material for: Medication-related knowledge and behavior profiles are generally favorable, but counseling needs remain among pregnant and postpartum women in Taiyuan, China: a cross-sectional study
Source: Front Public Health. 2026 Oct 16;14:1969907. doi: 10.3389/fpubh.2026.1969907 (PMC13719620; doi:10.3389/fpubh.2026.1969907)
Supplement: Supplementary file 1 [file Table_1.docx]

Supplementary Material

# 1 Supplementary Tables

Supplementary Table 1. Games–Howell post hoc pairwise comparisons of medication-knowledge risk scores by monthly income and occupation

**Panel A. Monthly income (Welch F(3, 237.04) = 3.449, P = 0.017)**

| **Pairwise comparison** | **Group means (x̄ ± s)** | **Mean difference (95% CI)** | **Adjusted P value** |
| --- | --- | --- | --- |
| Below 2,000 yuan (n = 84) vs 2,000–4,000 yuan (n = 167) | 63.20 ± 20.86 vs 59.18 ± 16.54 | 4.02 (-2.77 to 10.81) | 0.416 |
| Below 2,000 yuan (n = 84) vs 4,000–6,000 yuan (n = 143) | 63.20 ± 20.86 vs 61.45 ± 22.32 | 1.75 (-5.88 to 9.39) | 0.933 |
| Below 2,000 yuan (n = 84) vs Above 6,000 yuan (n = 101) | 63.20 ± 20.86 vs 55.56 ± 15.05 | 7.64 (0.56 to 14.72) | 0.029 |
| 2,000–4,000 yuan (n = 167) vs 4,000–6,000 yuan (n = 143) | 59.18 ± 16.54 vs 61.45 ± 22.32 | -2.27 (-8.12 to 3.58) | 0.748 |
| 2,000–4,000 yuan (n = 167) vs Above 6,000 yuan (n = 101) | 59.18 ± 16.54 vs 55.56 ± 15.05 | 3.62 (-1.48 to 8.71) | 0.260 |
| 4,000–6,000 yuan (n = 143) vs Above 6,000 yuan (n = 101) | 61.45 ± 22.32 vs 55.56 ± 15.05 | 5.88 (-0.31 to 12.07) | 0.069 |

**Panel B. Occupation (Welch F(8, 76.93) = 2.322, P = 0.027)**

| **Pairwise comparison** | **Group means (x̄ ± s)** | **Mean difference (95% CI)** | **Adjusted P value** |
| --- | --- | --- | --- |
| Enterprise Worker (n = 85) vs Company Employee (n = 101) | 59.04 ± 20.13 vs 60.77 ± 21.21 | -1.74 (-11.27 to 7.80) | 1.000 |
| Enterprise Worker (n = 85) vs Government Official (n = 21) | 59.04 ± 20.13 vs 55.67 ± 11.79 | 3.37 (-7.55 to 14.28) | 0.984 |
| Enterprise Worker (n = 85) vs Medical Institution Staff (n = 64) | 59.04 ± 20.13 vs 56.14 ± 17.71 | 2.89 (-6.90 to 12.69) | 0.991 |
| Enterprise Worker (n = 85) vs Teacher (n = 38) | 59.04 ± 20.13 vs 57.29 ± 19.53 | 1.75 (-10.55 to 14.04) | 1.000 |
| Enterprise Worker (n = 85) vs Enterprise Management (n = 19) | 59.04 ± 20.13 vs 52.58 ± 11.51 | 6.46 (-4.69 to 17.60) | 0.627 |
| Enterprise Worker (n = 85) vs Freelancer (n = 62) | 59.04 ± 20.13 vs 59.85 ± 17.13 | -0.82 (-10.53 to 8.89) | 1.000 |
| Enterprise Worker (n = 85) vs Student (n = 6) | 59.04 ± 20.13 vs 60.83 ± 11.77 | -1.80 (-23.93 to 20.34) | 1.000 |
| Enterprise Worker (n = 85) vs Others (n = 99) | 59.04 ± 20.13 vs 64.86 ± 19.27 | -5.82 (-14.99 to 3.35) | 0.549 |
| Company Employee (n = 101) vs Government Official (n = 21) | 60.77 ± 21.21 vs 55.67 ± 11.79 | 5.11 (-5.67 to 15.88) | 0.834 |
| Company Employee (n = 101) vs Medical Institution Staff (n = 64) | 60.77 ± 21.21 vs 56.14 ± 17.71 | 4.63 (-4.99 to 14.26) | 0.847 |
| Company Employee (n = 101) vs Teacher (n = 38) | 60.77 ± 21.21 vs 57.29 ± 19.53 | 3.48 (-8.69 to 15.66) | 0.991 |
| Company Employee (n = 101) vs Enterprise Management (n = 19) | 60.77 ± 21.21 vs 52.58 ± 11.51 | 8.19 (-2.82 to 19.20) | 0.297 |
| Company Employee (n = 101) vs Freelancer (n = 62) | 60.77 ± 21.21 vs 59.85 ± 17.13 | 0.92 (-8.62 to 10.46) | 1.000 |
| Company Employee (n = 101) vs Student (n = 6) | 60.77 ± 21.21 vs 60.83 ± 11.77 | -0.06 (-22.22 to 22.10) | 1.000 |
| Company Employee (n = 101) vs Others (n = 99) | 60.77 ± 21.21 vs 64.86 ± 19.27 | -4.09 (-13.07 to 4.90) | 0.886 |
| Government Official (n = 21) vs Medical Institution Staff (n = 64) | 55.67 ± 11.79 vs 56.14 ± 17.71 | -0.47 (-11.46 to 10.51) | 1.000 |
| Government Official (n = 21) vs Teacher (n = 38) | 55.67 ± 11.79 vs 57.29 ± 19.53 | -1.62 (-14.78 to 11.54) | 1.000 |
| Government Official (n = 21) vs Enterprise Management (n = 19) | 55.67 ± 11.79 vs 52.58 ± 11.51 | 3.09 (-9.03 to 15.21) | 0.995 |
| Government Official (n = 21) vs Freelancer (n = 62) | 55.67 ± 11.79 vs 59.85 ± 17.13 | -4.19 (-15.11 to 6.73) | 0.943 |
| Government Official (n = 21) vs Student (n = 6) | 55.67 ± 11.79 vs 60.83 ± 11.77 | -5.17 (-27.31 to 16.98) | 0.982 |
| Government Official (n = 21) vs Others (n = 99) | 55.67 ± 11.79 vs 64.86 ± 19.27 | -9.19 (-19.67 to 1.29) | 0.128 |
| Medical Institution Staff (n = 64) vs Teacher (n = 38) | 56.14 ± 17.71 vs 57.29 ± 19.53 | -1.15 (-13.51 to 11.21) | 1.000 |
| Medical Institution Staff (n = 64) vs Enterprise Management (n = 19) | 56.14 ± 17.71 vs 52.58 ± 11.51 | 3.56 (-7.65 to 14.78) | 0.980 |
| Medical Institution Staff (n = 64) vs Freelancer (n = 62) | 56.14 ± 17.71 vs 59.85 ± 17.13 | -3.71 (-13.51 to 6.08) | 0.956 |
| Medical Institution Staff (n = 64) vs Student (n = 6) | 56.14 ± 17.71 vs 60.83 ± 11.77 | -4.69 (-26.82 to 17.44) | 0.987 |
| Medical Institution Staff (n = 64) vs Others (n = 99) | 56.14 ± 17.71 vs 64.86 ± 19.27 | -8.72 (-17.98 to 0.55) | 0.083 |
| Teacher (n = 38) vs Enterprise Management (n = 19) | 57.29 ± 19.53 vs 52.58 ± 11.51 | 4.71 (-8.62 to 18.04) | 0.965 |
| Teacher (n = 38) vs Freelancer (n = 62) | 57.29 ± 19.53 vs 59.85 ± 17.13 | -2.57 (-14.86 to 9.73) | 0.999 |
| Teacher (n = 38) vs Student (n = 6) | 57.29 ± 19.53 vs 60.83 ± 11.77 | -3.54 (-25.75 to 18.66) | 0.999 |
| Teacher (n = 38) vs Others (n = 99) | 57.29 ± 19.53 vs 64.86 ± 19.27 | -7.57 (-19.47 to 4.34) | 0.523 |
| Enterprise Management (n = 19) vs Freelancer (n = 62) | 52.58 ± 11.51 vs 59.85 ± 17.13 | -7.28 (-18.42 to 3.87) | 0.469 |
| Enterprise Management (n = 19) vs Student (n = 6) | 52.58 ± 11.51 vs 60.83 ± 11.77 | -8.25 (-30.42 to 13.91) | 0.828 |
| Enterprise Management (n = 19) vs Others (n = 99) | 52.58 ± 11.51 vs 64.86 ± 19.27 | -12.28 (-23.01 to -1.55) | 0.015 |
| Freelancer (n = 62) vs Student (n = 6) | 59.85 ± 17.13 vs 60.83 ± 11.77 | -0.98 (-23.12 to 21.17) | 1.000 |
| Freelancer (n = 62) vs Others (n = 99) | 59.85 ± 17.13 vs 64.86 ± 19.27 | -5.00 (-14.18 to 4.17) | 0.735 |
| Student (n = 6) vs Others (n = 99) | 60.83 ± 11.77 vs 64.86 ± 19.27 | -4.03 (-26.26 to 18.21) | 0.994 |

Note: All pairwise comparisons are presented. Mean differences were calculated as the first group minus the second group. P values and 95% confidence intervals were adjusted using the Games–Howell procedure. Bold values indicate P < 0.05. Higher medication-knowledge risk scores indicate greater medication-related knowledge risk.

Supplementary Table 2. Exploratory structural validity and internal consistency of the medication-related KAP questionnaire

| **Domain** | **Items** | **n** | **KMO** | **Bartlett’s test, χ² (df)** | **Factors retained by parallel analysis** | **One-factor RMSR** | **Retained-factor RMSR** | **Cronbach’s α** |
| --- | --- | --- | --- | --- | --- | --- | --- | --- |
| Knowledge-risk | Q1–Q28 | 495 | 0.937 | 12,606.17 (378), P<0.001 | 3 | 0.085 | 0.042 | 0.957 |
| Behavior-risk | Q29–Q52 | 496 | 0.868 | 7,593.41 (276), P<0.001 | 4 | 0.182 | 0.040 | 0.808 |
| Attitude-related | Q53–Q63 | 497 | 0.868 | 6,797.00 (55), P<0.001 | 2 | 0.292 | 0.037 | 0.895 |

Supplementary Table 3. Two-factor structure of the attitude-related items

| **Subdomain** | **Items** | **Content** | **Main standardized loading range** | **Cronbach’s α** | **McDonald’s ω** |
| --- | --- | --- | --- | --- | --- |
| Exposure to medication education | Q53–Q58 | Frequency of exposure to lectures, educational materials, pharmacist consultation, and online medication information | 0.585–0.945 | 0.925 | 0.946 |
| Perceived necessity of medication education | Q59–Q63 | Perceived necessity of rational-medication lectures and educational materials | 0.830–0.978 | 0.948 | 0.963 |

# 2 English translation of the questionnaire administered in the study

**KAP Survey Questionnaire on Medication-Use Behavior Risk among Chinese Residents**

Hello! Thank you very much for participating in this KAP survey on medication-use behavior risk among Chinese residents. This survey is anonymous. Your responses will be used only by the researchers for statistical analysis, and there are no right or wrong answers. Your participation will provide meaningful support for promoting safe medication use among the public in China. Thank you very much for your support!

**Basic Information**

Before answering, please provide the following information (tick “√” next to the appropriate option):

| **Sex** | □ Male □ Female |
| --- | --- |
| **Age** | □ 19–34 years □ 35–49 years □ 50–64 years □ 65 years or older |
| **Monthly income** | □ Below 1,000 yuan □ 1,000–2,000 yuan □ 2,000–4,000 yuan □ 4,000–6,000 yuan □ Above 6,000 yuan |
| **Residential area** | □ Urban resident □ Rural resident |
| **Medical insurance status** | □ Basic social medical insurance □ Commercial insurance □ Self-payment □ Government-funded medical care □ Other |
| **Education level** | □ Postgraduate □ Bachelor’s degree □ College degree □ Technical secondary school or senior high school □ Junior high school □ Primary school |
| **Employment status** | □ Employed □ Retired □ Unemployed |
| **Occupation** | □ Enterprise worker □ Company employee □ Government official □ Medical institution staff □ Teacher □ Enterprise management □ Freelancer □ Student □ Other |

**Q1. Views and Opinions about Medications and Medication Use**

Each of the following statements presents a view or opinion about medications and medication use. Please indicate the extent to which you agree with each statement by ticking the corresponding number. A higher number indicates a greater degree of agreement. Please select one response for every item.

**1 = Strongly disagree**	**2 = Disagree**	**3 = Neutral**	**4 = Agree**	**5 = Strongly agree**

| **No.** | **Statement** | **1** | **2** | **3** | **4** | **5** |
| --- | --- | --- | --- | --- | --- | --- |
| 1 | When a person becomes ill, injections or intravenous infusions should be used whenever possible. | □ | □ | □ | □ | □ |
| 2 | Injectable medications, such as injections and intravenous infusions, are safer than oral medications. | □ | □ | □ | □ | □ |
| 3 | The more expensive a medication is, the safer it is. | □ | □ | □ | □ | □ |
| 4 | The more expensive a medication is, the more effective it is. | □ | □ | □ | □ | □ |
| 5 | The more types of medications used, the better the therapeutic effect. | □ | □ | □ | □ | □ |
| 6 | The longer the course of medication treatment, the better. | □ | □ | □ | □ | □ |
| 7 | Medication should be used immediately whenever even minor physical discomfort occurs. | □ | □ | □ | □ | □ |
| 8 | Health supplements are medications. | □ | □ | □ | □ | □ |
| 9 | Taking health supplements can reduce the amount of medication needed. | □ | □ | □ | □ | □ |
| 10 | Oral medications can be taken with milk, coffee, or other beverages. | □ | □ | □ | □ | □ |
| 11 | Nutritional health supplements can be taken at the same time as medications. | □ | □ | □ | □ | □ |
| 12 | When receiving an intravenous infusion in a hospital, the infusion time can be shortened at one’s own discretion. | □ | □ | □ | □ | □ |
| 13 | Antibacterial drugs can cure any type of cold or fever. | □ | □ | □ | □ | □ |
| 14 | Antibacterial drugs can kill all bacteria and viruses. | □ | □ | □ | □ | □ |
| 15 | As long as I do not misuse antibacterial drugs myself, antimicrobial resistance will not develop. | □ | □ | □ | □ | □ |
| 16 | The more types of antibacterial drugs taken at the same time, the faster the illness will improve. | □ | □ | □ | □ | □ |
| 17 | Antibacterial drugs are the same as anti-inflammatory drugs. | □ | □ | □ | □ | □ |
| 18 | Over-the-counter medications do not cause any adverse reactions. | □ | □ | □ | □ | □ |
| 19 | Unused medications should be stored in a refrigerator whenever possible. | □ | □ | □ | □ | □ |
| 20 | Medication can be discontinued when symptoms seem to improve. | □ | □ | □ | □ | □ |
| 21 | The medication dose can be reduced when symptoms seem to improve. | □ | □ | □ | □ | □ |
| 22 | The medication dose can be increased when symptoms seem to worsen. | □ | □ | □ | □ | □ |
| 23 | The type of medication can be changed when symptoms seem to worsen. | □ | □ | □ | □ | □ |
| 24 | New medications are definitely more effective and safer than older medications. | □ | □ | □ | □ | □ |
| 25 | When people around me develop a cold, fever, or another illness, antibacterial drugs can be taken to prevent illness. | □ | □ | □ | □ | □ |
| 26 | When purchasing medications, the quality of a medication should be judged by its brand and reputation. | □ | □ | □ | □ | □ |
| 27 | All medications can be stored at room temperature. | □ | □ | □ | □ | □ |
| 28 | When purchasing medications, price is unimportant; the key consideration is good therapeutic efficacy. | □ | □ | □ | □ | □ |

**Q2. Medication-Use Behaviors**

The following statements describe behaviors that may occur during medication use. Please indicate the frequency with which each behavior actually occurs. A higher number indicates a higher frequency.

**1 = Never**	**2 = Occasionally**	**3 = Sometimes**	**4 = Often**	**5 = Always**

**Q2-1. Before using medications**

| **No.** | **Behavior** | **1** | **2** | **3** | **4** | **5** |
| --- | --- | --- | --- | --- | --- | --- |
| 29 | I purchase medications on my own based on my previous experience or advertisements. | □ | □ | □ | □ | □ |
| 30 | When seeing a doctor, I specifically ask the doctor to prescribe a particular medication. | □ | □ | □ | □ | □ |
| 31 | When purchasing medications at a pharmacy, I follow the recommendations of pharmacy sales staff. | □ | □ | □ | □ | □ |
| 32 | I take a physician’s prescription to a pharmacy when purchasing prescription medications. | □ | □ | □ | □ | □ |
| 33 | I regularly check the medications stored at home. | □ | □ | □ | □ | □ |
| 34 | I keep medications in places that are easily accessible to children. | □ | □ | □ | □ | □ |
| 35 | I check the storage conditions stated in the medication instructions. | □ | □ | □ | □ | □ |
| 36 | I check the adverse effects listed in the medication instructions and the manifestations that may occur if adverse effects develop. | □ | □ | □ | □ | □ |
| 37 | I learn about precautions or contraindications when using a medication, such as alcohol consumption or driving. | □ | □ | □ | □ | □ |
| 38 | I check the expiration date before taking a medication. | □ | □ | □ | □ | □ |
| 39 | Before taking a medication, I check the drug approval number printed on the package. | □ | □ | □ | □ | □ |

**Q2-2. While using medications**

| **No.** | **Behavior** | **1** | **2** | **3** | **4** | **5** |
| --- | --- | --- | --- | --- | --- | --- |
| 40 | I throw expired medications stored at home into the household trash. | □ | □ | □ | □ | □ |
| 41 | I forget to take or use medications during a course of treatment. | □ | □ | □ | □ | □ |
| 42 | When a child refuses to take medicine, I pinch the child’s nose and force the medicine into his or her mouth. | □ | □ | □ | □ | □ |
| 43 | I take medications that have passed their expiration date. | □ | □ | □ | □ | □ |
| 44 | I take medications together with milk, tea, or coffee. | □ | □ | □ | □ | □ |
| 45 | Whenever I use medication, I take my own physical condition into consideration. | □ | □ | □ | □ | □ |
| 46 | I increase the medication dose on my own to improve its effect or when my symptoms worsen. | □ | □ | □ | □ | □ |
| 47 | I do not pay attention to the recommended time intervals between medication doses. | □ | □ | □ | □ | □ |
| 48 | If I feel that a medication has not worked after taking it for several days, I change to another medication on my own. | □ | □ | □ | □ | □ |
| 49 | After my condition improves, I reduce the dose or discontinue the medication on my own. | □ | □ | □ | □ | □ |
| 50 | While taking Western medicine, I also choose to take traditional Chinese medicine on my own. | □ | □ | □ | □ | □ |
| 51 | I split or crush tablets before taking them. | □ | □ | □ | □ | □ |
| 52 | Without guidance from a physician or pharmacist, I take several different medications together. | □ | □ | □ | □ | □ |

**Q3. Exposure to Rational Medication-Use Education**

The following statements describe medication-related lectures or educational activities that you may have encountered. Please indicate the frequency with which you have actually been exposed to or participated in these activities.

**1 = Never**	**2 = Occasionally**	**3 = Sometimes**	**4 = Often**	**5 = Always**

| **No.** | **Educational activity** | **1** | **2** | **3** | **4** | **5** |
| --- | --- | --- | --- | --- | --- | --- |
| 53 | I attend lectures on rational medication use in the community where I live. | □ | □ | □ | □ | □ |
| 54 | I attend lectures on rational medication use at hospitals or community health service centers. | □ | □ | □ | □ | □ |
| 55 | I read educational materials on rational medication use distributed by the community residents’ committee. | □ | □ | □ | □ | □ |
| 56 | I receive pharmacist consultation services in the community or at public locations. | □ | □ | □ | □ | □ |
| 57 | I read educational materials on rational medication use distributed by hospitals or community health service centers. | □ | □ | □ | □ | □ |
| 58 | I read educational materials on rational medication use through the Internet, WeChat, or similar platforms. | □ | □ | □ | □ | □ |

**Q4. Perceived Necessity of Rational Medication-Use Education**

If you have been exposed to any of the above forms of education on rational medication use, please indicate how necessary you believe these activities are.

**1 = Completely unnecessary**	**2 = Unnecessary**	**3 = Neutral**	**4 = Necessary**	**5 = Very necessary**

| **No.** | **Educational activity** | **1** | **2** | **3** | **4** | **5** |
| --- | --- | --- | --- | --- | --- | --- |
| 59 | Community lectures on rational medication use. | □ | □ | □ | □ | □ |
| 60 | Lectures on rational medication use at hospitals or community health service centers. | □ | □ | □ | □ | □ |
| 61 | Educational materials on rational medication use distributed by community residents’ committees. | □ | □ | □ | □ | □ |
| 62 | Educational materials on rational medication use distributed by hospitals or community health service centers. | □ | □ | □ | □ | □ |
| 63 | Educational materials on rational medication use disseminated through the Internet, WeChat, or similar platforms. | □ | □ | □ | □ | □ |

**Q5. Medication Use during Pregnancy and the Postpartum Period**

The following questions are intended for pregnant and postpartum women and their family members. Please answer according to your actual situation.

**1 = Very unfamiliar**	**2 = Unfamiliar**	**3 = Generally familiar**	**4 = Very familiar**	**5 = Extremely familiar**

| **No.** | **Question** | **1** | **2** | **3** | **4** | **5** |
| --- | --- | --- | --- | --- | --- | --- |
| 64 | Do you understand the risk classification of drug use during pregnancy and lactation? | □ | □ | □ | □ | □ |
| 65 | Do you have knowledge about medications for gestational hypertension and gestational hyperglycemia? | □ | □ | □ | □ | □ |
| 66 | Do you understand the relevant medication use during the postpartum lactation period? | □ | □ | □ | □ | □ |
| 67 | What do you know about medications for postpartum lactation suppression? | □ | □ | □ | □ | □ |
| 68 | Do you understand postpartum analgesic medications? | □ | □ | □ | □ | □ |
